# Whole genome QTL mapping for growth, meat quality and breast meat yield traits in turkey

**DOI:** 10.1186/1471-2156-12-61

**Published:** 2011-07-11

**Authors:** Muhammad L Aslam, John WM Bastiaansen, Richard PMA Crooijmans, Addie Vereijken, Martien AM Groenen

**Affiliations:** 1 Animal Breeding and Genomics Centre, Wageningen University, 6709PG, Wageningen, the Netherlands; 2 Hendrix Genetics, Research & Technology Centre, 5830 AC, Boxmeer, the Netherlands

## Abstract

**Background:**

The turkey (*Meleagris gallopavo*) is an important agricultural species and is the second largest contributor to the world's poultry meat production. Demand of turkey meat is increasing very rapidly. Genetic markers linked to genes affecting quantitative traits can increase the selection response of animal breeding programs. The use of these molecular markers for the identification of quantitative trait loci, and subsequently fine-mapping of quantitative trait loci regions, allows for pinpointing of genes that underlie such economically important traits.

**Results:**

The quantitative trait loci analyses of the growth curve, body weight, breast yield and the meat quality traits showed putative quantitative trait loci on 21 of the 27 turkey chromosomes covered by the linkage map. Forty-five quantitative trait loci were detected across all traits and these were found in 29 different regions on 21 chromosomes. Out of the 45 quantitative trait loci, twelve showed significant (p < 0.01) evidence of linkage while the remaining 33 showed suggestive evidence (p < 0.05) of linkage with different growth, growth curve, meat quality and breast yield traits.

**Conclusion:**

A large number of quantitative trait loci were detected across the turkey genome, which affected growth, breast yield and meat quality traits. Pleiotropic effects or close linkages between quantitative trait loci were suggested for several of the chromosomal regions. The comparative analysis regarding the location of quantitative trait loci on different turkey, and on the syntenic chicken chromosomes, along with their phenotypic associations, revealed signs of functional conservation between these species.

## Background

The turkey (*Meleagris gallopavo*, MGA) is an important agricultural species and is the second largest contributor to the world's poultry meat production. Turkey stocks increased from 178 to 482 million and production volume increased from 1.2 to 5.6 M. tons between 1970 to 2008 [1]. This rapidly increasing demand of turkey meat motivated breeders and farmers to produce rapidly growing birds with a high market body weight (BW) and a desirable body conformation in order to maximize production efficiency and optimize production of preferred body cuts; e.g., breast muscle yield [2].

Commonly applied breeding programs for meat type birds, select for body weight (BW) and body composition traits (breast muscle yield, etc.), while minimizing production costs. Recently, breeders have started measuring meat quality traits (drip loss, pH and color) as well as survival traits, at least in research project settings [3,4]. Selection efforts have improved BW and body composition (i.e. increasing breast yield and lowering carcass fatness). These improvements, however, have also led to indirect and sometimes deleterious effects on meat quality and fitness traits [3]. Genetic parameters (heritabilities, genetic and phenotypic correlations) for the growth, meat quality and breast yield traits in turkey birds have been estimated [5], and showed unfavorable correlations of meat quality traits with the growth and the breast yield traits. The use of molecular markers that are directly or indirectly linked to QTL could provide potent tools to overcome these challenging correlations [6,7]. In addition, identification and subsequent fine-mapping of QTL regions should allow for the pinpointing of genes that underlie such traits.

Several studies have indicated that knowledge about genetic markers linked to genes affecting quantitative traits can increase the selection response of animal breeding programs, especially for traits that are difficult to improve by traditional selection [8,9]. Significant association between individual genetic markers and quantitative traits of economic importance have been reported in chicken [10-13] but no such reports exist for turkey.

A large number of studies are available on QTL mapping for the growth, meat quality and the body composition traits of chicken [7,11,14-16] showing significant effects of QTLs on these traits of economic importance in poultry breeding.

The detection of QTL and exploration of the underlying genes controlling these traits will benefit poultry breeding programs [17]. With this study we aim to build the same potential for turkey breeding programs by detecting quantitative trait loci for growth, meat quality and breast yield traits in turkey.

## Methods

### Resource population

Parents were randomly selected from two different commercial lines of turkey to produce F1 offspring [18]. Ten parent males were randomly selected from a high growth male line that contributed to a "large white product". Ten parent females were randomly selected from a high reproduction female line that contributed to a "heavy medium product". Average BW of males in the high growth line was 11.5 Kg and the average body weight of males in high reproduction line was 7.4 Kg at 14 weeks of age. Average egg production in the high growth line was 59.3 hatching eggs/24 weeks while average egg production in the high reproduction line was 115.5 hatching eggs/24 weeks. Parents were crossed to produce 10 full-sib families in the F1 generation. An F2 generation of 18 full sib families was produced by crossing 17 randomly selected F1 males and 18 randomly selected F1 females. One of the males was mated with two females; other F1 parents were mated only once. The F2 individuals were from 14 different hatches. In total, 973 F2 offspring were produced with an average full sib family size of 54.1 and a range of 31 to 90 individuals per family.

### Traits

Phenotypic data were recorded within a commercial breeding program. Body weight (BW), breast yield (BrY) and meat quality (MQ) traits were recorded on individuals of the F2 generation. Body weights were recorded at 1, 17, 40, 60, 80, and 120 days (BW01, BW17, BW40, BW60, BW80, and BW120, respectively). The breast meat yield traits; breast length (BrL), breast width (BrW), percent breast meat (PBM, *Pectoralis *(*P*) major and *P*. minor) and meat quality traits; percent drip loss (PDL), ultimate pH (pHu) and breast meat color (CIE L*a*b* system, where L* represents lightness, a* redness and b* yellowness) were measured at 20 weeks of age. These traits were measured as described previously [5].

Body weight observations at different time points were used to derive logistic growth curve traits i.e. asymptotic weight (As_wt_), inflection point at which 50% of the asymptotic weight is achieved (t_mid_), and a constant that is proportional to the overall growth rate (scale). The procedures and methodology for the estimation of these traits have previously been described [5].

### Genotype data and linkage map

The marker data and the linkage map utilized in the study were described in Aslam et al. [18]. The genotype data of 522 SNP, mapped to 27 turkey autosomes, was available after removal of uninformative and problematic SNP from the total set of 775 SNP [18]. The sex average linkage map was used, which had a length of 2164.8 cM with an average marker spacing of 4.4 cM. The data also included SNP that were specifically selected from 5 different turkey genes; *PIT1*, *AFABP*, *PRKAG3*, *IGF2 *and *GDF8*.

### Statistical analysis

#### Descriptive analysis

Basic descriptive statistics, including number of observations (N), minimum values, maximum values, means and standard deviations (s.d.) were calculated by PROC MEANS of SAS software [19]. Fixed effects of sex and hatch were tested for significance on each trait with PROC GLM [19]. Effects that were found to be significant (P < 0.05) were included in the model for the QTL mapping analysis.

#### QTL mapping

A regression-interval mapping method was applied which is available through the web-based software QTL EXPRESS accessed via the GridQTL portlet [20]. GridQTL is a portlet environment (available at http://www.gridqtl.org.uk/) that permits the analysis of computationally intensive datasets. Because of the full-sib structure in the F2, and the absence of genotypes on the parent generation, the analyses were carried out by applying a sib-pair model. Sex and hatch (n = 14) effects were tested for all traits and included in the model only if statistically significant (P < 0.05).

F-statistic profiles were generated at 1 cM intervals along each chromosome to identify the most likely QTL position. Significance thresholds were determined by permutation of the dataset [21], with 10,000 permutations performed to obtain single position as well as chromosome-wide significance levels. QTL that exceeded the chromosome-wide F-critical threshold at a P < 0.05 were reported as suggestive QTL, while exceeding a chromosome-wide F-critical threshold of P < 0.01 was considered evidence for a significant QTL effect. QTL variance estimates were obtained from a separate regression analysis of squared differences on IBD sharing of full-sibs at the QTL positions [22].

On each chromosome, regions were defined based on the occurrence of QTL. Two or more QTL were considered to be located in the same region if the distance between the chromosomal positions of these QTL was equal or less than 10 cM.

#### Comparative QTL mapping

All significant as well as all suggestive turkey QTL were mirrored on the chicken genome. Nucleotide positions of SNP flanking the turkey QTL were mapped to chicken chromosomes and the chicken nucleotide positions were subsequently used to obtain cM positions on the chicken genome [18] that correspond to the positions of QTL discovered in turkey. These chicken genome positions of turkey QTL were compared to chicken QTL positions for the same trait, or a very similar trait, which were obtained from QTLdb [23]. The distance of the turkey QTL position on the chicken map to the nearest chicken QTL for the same trait was calculated.

To test whether QTL are conserved between chicken and turkey we used the distance from a random chicken map position to a chicken QTL as our null hypothesis. Under the null hypothesis, chicken linkage map positions (cM) were randomly chosen (n = 100) and their average distance to BW QTL from the chicken QTLdb was calculated. The distance between randomly selected positions from the chicken linkage map and the nearest QTL position from QTLdb were averaged and compared to the average distance between chicken and turkey QTL for the same trait.

#### Ethical approval for the use of animals in this study

Although animals were used in this experimental work, no direct experiments were performed on them. Blood sample collection was carried out by licensed and authorized personnel under approval of Hendrix Genetics. No approval from the ethics committee was necessary.

## Results

### Descriptive Analysis

A descriptive analysis of all the traits under study is summarized in Table 1. The effect of sex was significant (P < 0.0005) for all the traits except for the weight of 1 day old chicks (BW01), percent breast meat (PBM) and the redness of meat (a*). The effect of hatch was also significant for all the traits.

**Table 1:** Descriptive statistics, including the estimates for the significant fixed effects (Sex and Hatch).

Traits(units)	N	Minimum	Maximum	LS Mean	RSD	**Sex**^1^	**Hatch**^2^
**BW01(Kg)**	810	0.04	0.07	0.06	0.06	0.00	0.02*
**BW17(Kg)**	785	0.08	0.60	0.33	0.43	0.22*	0.13*
**BW40(Kg)**	751	0.52	2.32	1.35	1.64	0.69*	0.31*
**BW60(Kg)**	710	1.50	4.96	3.11	3.65	1.27*	0.55*
**BW80(Kg)**	693	3.06	8.50	5.45	6.33	2.25*	1.19*
**BW120(Kg)**	655	4.54	15.90	10.39	12.19	5.04*	1.50*
**PBM (%)**	785	0.02	13.40	10.73	2.15	0.10	8.83*
**BrL(mm)**	937	155.00	300.00	212.57	28.53	48.30*	21.29*
**BrW(mm)**	937	109.00	203.00	146.88	16.17	25.68*	21.39*
**PDL (%)**	828	2.21	14.10	5.09	1.28	0.94*	1.35*
**pHu**	838	5.26	6.02	5.75	0.11	0.04*	0.53*
L*	864	40.30	53.60	45.92	1.82	0.98*	2.65*
a*	864	1.30	9.20	5.27	1.00	0.09	2.56*
b*	864	0.10	5.60	2.28	0.84	0.54*	0.63*
**As_wt_(Kg)**	645	4.65	20.23	12.29	3.47	6.50*	2.92*
**T_mid_(Day)**	645	59.86	112.24	82.85	5.44	6.14*	11.75*
**Scale(Day)**	645	12.66	29.15	20.61	2.03	1.95*	5.13*

N = Number of records; minimum = minimum values; maximum = maximum values; LS Mean = least square mean; RSD = residual standard deviation; BW01, BW17, BW40, BW60, BW80, and BW120 are the BW at days 1,17, 40, 60, 80, and 120 of age, respectively; PBM = percentage breast meat at 20 week of age; BrL = breast length at 20 week of age; BrW = breast width at 20 wk of age; PDL = percent drip loss at 20 week of age; pHu = ultimate pH at 20 wk of age; L* = lightness at 20 wk of age; a* = redness at 20 wk of age; b* = yellowness at 20 wk of age; As_wt _= upper asymptotic weight (estimated growth curve parameter); t_mid _= inflection point at 50% asymptote (estimated growth curve parameter); scale = constant that is proportional to the overall growth rate (estimated growth curve parameter). ^1 ^= Difference between sexes in the Least square means (LS Means) of the traits. ^2 ^= Difference between the maximum and minimum LS Means of the traits with respect to the week of hatch.

*P ≤ 0.0005

### QTL mapping

QTL that surpassed the suggestive or significant linkage threshold were summarized in Tables 2, 3, 4 &5. The QTL analyses for the growth curve (Table 2), BW (Table 3), BY (Table 4) and the MQ traits (Table 5) showed putative QTL on 21 of the 27 turkey chromosomes covered by the linkage map. Forty-five QTL were detected across all traits and these were found in 29 different regions on 21 chromosomes. Out of the 45 QTL, twelve QTL showed significant (p < 0.01) evidence of linkage while the remaining 33 QTL showed suggestive evidence (p < 0.05) of linkage with different growth, growth curve, meat quality and breast yield traits.

**Table 2:** QTL mapped on different chromosomes of turkey affecting growth curve traits.

						F-Statistics Threshold^1^	
						
Trait	Chromosome	Location (cM)	qtlV	F-Statistics	Flanking Markers	P < 0.05	P < 0.01
**Scale**	MGA2	113	0.07	15.40	B002042-A004960	10.03	16.35
**As_wt_**	MGA3	92	0.09	11.91	A005884-A001055	10.53	17.82
**As_wt_**	MGA13	49	0.09	12.16*	A002976-B002771	6.67	11.04
**Scale**	MGA15	30	-0.07	14.59*	B002847-A003255	8.89	13.52
**Aswt**	MGA22	2	0.10	12.30	A000901-A006033	6.58	12.83
**T_mid_**	MGA22	6	-0.02	7.61	A003266-A000012	6.80	13.42
**Scale**	MGA22	5	0.05	10.91*	A006033-A003266	6.46	10.56
**As_wt_**	MGA28	16	0.17	18.70*	B000023-B001881	5.05	8.12

* QTLs with significant evidence (P < 0.01). ^1^Chromosome wide significance thresholds from permutation test.

**Table 3:** QTL mapped on different chromosomes of turkey affecting body weight traits.

						F-Statistics Threshold^1^	
						
Trait	Chromosome	Location (cM)	qtlV	F-Statistics	Flanking Markers	P < 0.05	P < 0.01
**BW40**	MGA1	217	0.03	11.05	B003270-A005799	10.81	15.82
**BW17**	MGA5	63	0.15	10.24	A001354-A005103	8.89	16.02
**BW40**	MGA5	60	0.11	10.40	A001354-A005103	9.22	15.26
**BW40**	MGA8	1	0.11	11.49	B000608-A001480	7.29	11.63
**BW60**	MGA8	1	0.06	9.05	B000608-A001480	7.17	11.51
**BW80**	MGA8	1	0.07	11.95*	B000608-A001480	7.29	11.62
**BW40**	MGA12	0	0.18	8.39	B000094-B000257	5.91	10.40
**BW80**	MGA12	1	0.13	8.47	B000094-B000257	6.26	10.98
**BW120**	MGA13	54	0.05	7.81	A002976-B002771	6.95	12.86
**BW40**	MGA20	51	0.04	7.93	B002015-B002517	6.73	11.78
**BW120**	MGA22	0	0.12	12.15	B002897-A000901	7.52	13.29
**BW40**	MGA22	6	0.08	8.78	A003266-A000012	7.60	12.39
**BW40**	MGA26	0	0.15	9.54	B000407-B002784	8.23	16.83
**BW120**	MGA28	12	0.11	11.04*	B000023-B001881	5.80	10.23
**BW01**	MGA30	0	-0.01	9.08*	B003031-B000504	4.39	9.07

* QTLs with significant evidence (P < 0.01). ^1^Chromosome wide significance thresholds from permutation test.

**Table 4:** QTL mapped on different chromosomes of turkey affecting breast yield traits.

						F-Statistics Threshold^1^	
						
Trait	Chromosome	Location (cM)	qtlV	F-Statistics	Flanking Markers	P < 0.05	P < 0.01
**BrW**	MGA3	132	0.15	16.64	B003202-B002875	10.87	16.67
**PBM**	MGA4	29	0.18	10.88	A006113- B001871	8.27	11.94
**BrL**	MGA5	113	0.06	9.19	A003231-A000813	8.57	14.63
**PBM**	MGA11	36	0.30	9.65	B002433-A003945	7.39	12.49
**PBM**	MGA19	41	0.14	7.31*	B002491-B002546	3.41	5.95
**PBM**	MGA22	6	0.22	10.49	A003266-A000012	7.78	12.24
**PBM**	MGA26	45	0.38	9.25	B002264-A006279	8.20	14.15
**BrL**	MGA28	4	-0.01	17.10*	B000278-B000023	5.27	9.32
**BrW**	MGA28	0	-0.01	5.86	B000278-B000023	5.13	8.26

* QTLs with significant evidence (P < 0.01). ^1^Chromosome wide significance thresholds from permutation test.

**Table 5:** QTL mapped on different chromosomes of turkey affecting meat quality traits.

						F-Statistics Threshold^1^	
						
Trait	Chromosome	Location (cM)	qtlV	F-Statistics	Flanking Markers	P < 0.05	P < 0.01
**PDL**	MGA1	71	0.17	15.57	B001935-B001936	10.44	15.90
b*	MGA3	107	0.07	16.66*	A002870-B003116	9.41	15.02
**PDL**	MGA3	65	0.11	10.61	B003023-B002640	8.91	12.62
b*	MGA4	30	0.1	9.88	B001871-B002284	8.72	13.46
**PDL**	MGA7	0	0.1	10.62	A001382-B002403	7.98	13.70
L*	MGA8	1	0.07	8.16	B000608-A001480	7.37	12.12
b*	MGA12	27	0.08	28.46*	A004841-A004198	6.44	9.87
**PDL**	MGA12	17	0.06	5.99	A001153-B000396	5.37	8.36
**PDL**	MGA14	55	0.08	9.39	A003474-B002743	7.38	12.29
**PDL**	MGA17	52	0.19	10.92	A003133-A000203	7.54	13.18
b*	MGA21	61	0.08	11.53	B003125-A004009	8.34	15.02
**PDL**	MGA24	30	0.06	7.11	B000536-B002896	5.41	10.38
b*	MGA26	43	0.1	17.06*	B002430-B002264	8.09	15.25

* QTLs with significant evidence (P < 0.01). ^1^Chromosome wide significance thresholds from permutation test.

MGA3 appeared to be important for all trait groups except BW traits, with four different regions affecting As_wt_, BrW, b* and PDL at 92, 132, 107 and 65 cM respectively (Table 2, 4 &5). The QTL for b* on chromosome 3 was found significant, the others were suggestive. The four QTL affected four different traits and their positions were also in different regions which suggests that four different QTL were involved, one for each of the traits.

**Figure 1:**
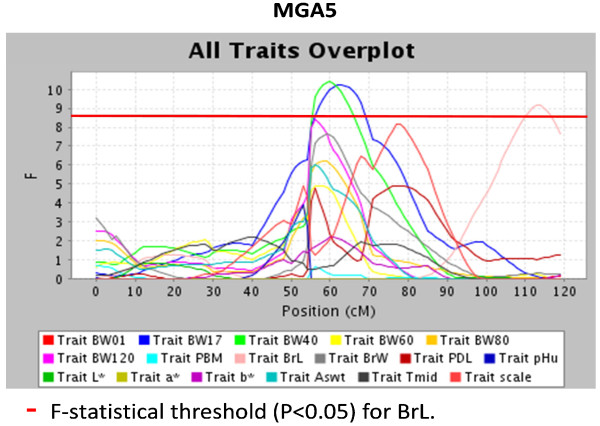
**Identified QTL on turkey chromosome 5 affecting growth, meat quality and breast yield traits**. BW01, BW17, BW40, BW60, BW80, and BW120 are the BW at days 1,17, 40, 60, 80, and 120 of age; PBM = percentage breast meat at 20 wk of age; BrL = breast length at 20 wk of age; BrW = breast width at 20 wk of age; PDL = percent drip loss at 20 wk of age; pHu = ultimate pH at 20 wk of age; L* = lightness at 20 wk of age; a* = redness at 20 wk of age; b* = yellowness at 20 wk of age; As_wt _= upper asymptote (estimated growth curve parameter); t_mid _= inflection point at 50% asymptote (estimated growth curve parameter); scale = constant that is proportional to the overall growth rate (estimated growth curve parameter).

**Figure 2:**
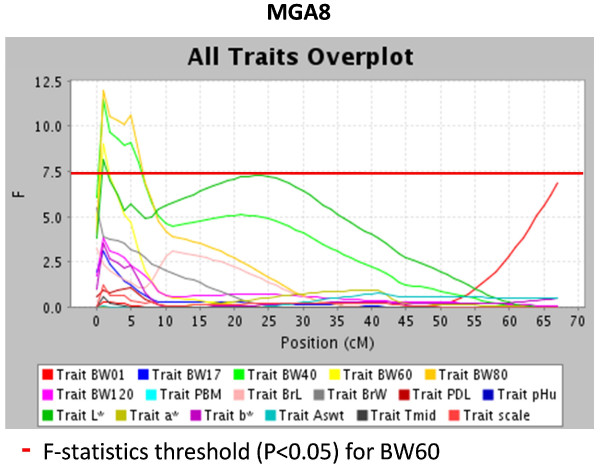
**Identified QTL on turkey chromosome 8 affecting growth, meat quality and breast yield traits**. BW01, BW17, BW40, BW60, BW80, and BW120 are the BW at days 1,17, 40, 60, 80, and 120 of age; PBM = percentage breast meat at 20 wk of age; BrL = breast length at 20 wk of age; BrW = breast width at 20 wk of age; PDL = percent drip loss at 20 wk of age; pHu = ultimate pH at 20 wk of age; L* = lightness at 20 wk of age; a* = redness at 20 wk of age; b* = yellowness at 20 wk of age; As_wt _= upper asymptote (estimated growth curve parameter); t_mid _= inflection point at 50% asymptote (estimated growth curve parameter); scale = constant that is proportional to the overall growth rate (estimated growth curve parameter).

Two QTL regions were detected on chromosome 5, the first region showed a QTL for development in weight (BW17 and BW40) at 60-63 cM, and the second region showed a QTL for BrL at 113 cM. The QTL for BrL was in a separate region. Another region with QTL for development in BW traits (BW40, BW60 and BW80) was located on chromosome 8 at cM position 1 (Figure 1 &2).

Two regions on MGA12, the first with QTL affecting weight development (BW40 and BW80) and the second with QTL affecting the quality of meat (b*, and PDL) were detected at 0 to 1 and 17 to 27 cM respectively (Table 3 &5).

In our study, MGA22 showed multiple QTL affecting growth (growth curve and BW traits) as well as a QTL with an effect on PBM. A QTL at position 0 to 6 cM showed significant evidence (p < 0.01) for an effect on the growth curve trait scale, while at the same position suggestive evidence (p < 0.05) was found for an effect on the other growth traits BW40, BW120, As_wt_, and t_mid _as well as an effect on PBM (Table 2, 3 &4).

Again, multiple QTL were detected on chromosome 28 with significant effects on As_wt_, BW120 and BrL and with suggestive evidence for BrW (Table 2, 3 &4) with QTL positions between 0 and 12 cM.

When focusing on meat quality, QTL with significant effects (p < 0.01) on meat quality, yellowness (b*), were detected on chromosome 3, 12 and 26 at position 107 cM, 27 cM and 43 cM respectively (Table 5). Additional QTL with suggestive effects on percent drip loss were detected on chromosome 1, 3 and 12 at position 71, 65 cM and 17 cM respectively (Table 5). Suggestive evidence of a QTL affecting lightness (L*) of meat was also detected on chromosome 8 at cM position 1 (Figure 2). No significant QTL was detected for redness (a*) and the ultimate pH (pHu) of meat (Additional file 1 &2).

### Comparative QTL mapping

For seven out of the 15 turkey QTL that affected BW traits, QTL were found for the same or a very similar trait on syntenic regions in the chicken genome, within a distance of 8 cM or less. The average distance between syntenic positions of the turkey BW QTL in chicken and the nearest chicken QTL positions (from QTLdb) was 14.7 cM (Additional file 3). The seven turkey QTL with nearby syntenic chicken QTL were detected on MGA1, 5, 13, 20 and MGA22. A turkey QTL affecting b* was also found nearby a chicken QTL for b* with a distance of less than 7 cM between the syntenic QTL positions in these species. This QTL for b* was detected on MGA12 (Additional file 3).

The distance from a randomly selected positions (n = 100) on the chicken linkage map to the nearest chicken QTL for BW traits was on average 18.06 ± 3.08 cM (Additional file 3).

## Discussion

QTL were detected for growth, breast yield and meat quality traits which are important traits in poultry breeding. This study adds important new information from a genome wide search for QTL in turkeys, and is the first to report the detection and positioning of loci affecting commercially important traits in turkeys.

Several chromosomes showed multiple QTL at nearby positions, indicating that pleiotropic effects may be playing a role. We expected to find overlapping QTL positions for multiple BW traits because these traits were previous found to have high genetic correlations among each other [5]. In the present study, eight QTL were detected with a significant effect on growth. For seven of these eight QTL, additional significant or suggestive QTL for other growth traits were detected in the same chromosome region. This presence of multiple QTL for genetically correlated traits suggests the presence of QTL with pleiotropic effects on these traits. A good example is the identification of QTL for As_wt _and BW120 in the same region of chromosomes 13, 22 and 28. Traits As_wt _and BW120 are very similar traits that both represent mature BW and have a high genetic correlation of nearly 1 [5].

Comparative studies of turkey and chicken based on cytogenetic [24], genome sequence [25], and linkage [18] analyses have shown highly conserved karyotypes and genomic structure between these species. In the present study, a number of traits were found to be affected by QTL on MGA22 including BW traits. MGA22 appeared to play a role in the genetic variation of growth patterns in turkey, harboring a QTL with an effect on all three growth curve traits (As_wt_, t_mid _and scale). QTL models were fitted on growth curve parameters to estimate effects on parameters that can be interpreted for their biologically meaning in the growth pattern, in addition to results from applying QTL models on BW observations at different time points. Applying QTL models on BW observations estimates the effect of a QTL on weight at that particular age while applying QTL model on growth curve parameters may give insight in the effect a of QTL throughout the growth pattern of an individual [26]. The QTL affecting the BW traits on chromosome 22 of turkey are located at a position syntenic to a region on GGA20 which was previously shown to contain a QTL for growth [15,27] (Additional file 3). Likewise, the region on MGA1 containing the QTL for PDL is syntenic to a region on GGA1 also shown to contain a QTL for the same trait [14].

The identification of QTLs affecting BW traits on MGA1, 5, 13, 20, MGA22 and a QTL affecting meat color trait (b*) on MGA12 are also in agreement with the QTL reported for these traits on the syntenic GGA1, 5, 11, 18, 20, and GGA10 respectively [27-30]. A high level of structural genomic conservation has been identified between turkey and chicken [18,24,25]. The comparison of turkey QTL positions, mirrored on the chicken genome, with the chicken QTL positions for the same trait suggests that in addition to the structural genomic conservation, functional genomic conservation also exist between these species.

The SNPs that are located within growth related genes (*PIT1*, *AFABP*, *PRKAG3*, *IGF2 *and *GDF8) *were used to test for direct effects of these SNPs on the growth traits. When these SNPs were included as fixed effects in the model, the F-value at the position of these SNPs decreased by more than 50%. The large impact of these SNPs on the QTL model does not necessarily mean that the SNPs are causative mutations, but these SNPs explain an important amount of QTL variation, either directly or through LD with the causative mutations. The candidate genes (*PIT1*, *AFABP*, *PRKAG3*, *IGF2 *and *GDF8) *were known to affect growth related traits in other species making it likely that these are the actual genes underlying the QTL effects, even though LD extends over large regions [31] and the other genes in the neighborhood cannot be excluded.

Estimates of QTL variance were not obtained from the QTLexpress analysis output. To estimate the variance explained by each QTL, the regression slopes were used to calculate QTL variances (qtlV) as a proportion of the residual variance. These estimates of QTL variance are likely to be overestimates [32], but for a few QTL a negative QTL variance estimate was obtained because the regression slopes were positive in the regressions used to estimate them.

To search for positional candidate genes near the QTL, the sequence annotation of turkey was used. The Positions (cM) of the SNPs flanking the significant turkey QTLs, as well as the sequence surrounding the SNPs, were used to convert the cM positions of QTL on the linkage map into base-pair (bp) positions on the turkey genome. First the sequences around SNPs that flank the turkey QTL were used to obtain the position (bp) of these SNPs in the turkey genome [18]. Subsequently, the approximate position (bp) of turkey QTL in the turkey genome was predicted by using the relative distances in cM of the turkey QTL to the flanking SNP positions. Then these same relative distances were applied to the interval between the turkey genome positions (bp) of the flanking SNPs. Finally, functional information was inspected for genes within a region of ± 500 kb from the predicted QTL positions (bp) for the 10 longest chromosomes and within ± 100 kb for the 20 smallest chromosomes. Near most QTL, genes were found with unknown function or functions related to metabolism or transcription and translation processes. These genes can be responsible for the QTL effects that were found but no conclusion can be drawn. No genes were found on MGA22 within the window of ± 100 kb from the QTL position (bp) (Additional file 4).

As described earlier, the turkey QTL positions (bp) were mirrored onto the chicken genome. Genes on the chicken genome were identified within the same window ranges as applied in turkey. Two potential candidate genes were found in chicken for turkey QTL, namely EYA1 and Col5A1 which have functions in morphogenesis (drosophila) [33,34] and fibrillogenesis [35] respectively. The genes EYA1 and Col5A1 were present in the syntenic turkey chromosomes but were positioned at 1345 kb and 300.4 Kb away from the QTL positions (bp) in the turkey genome which were outside of selected search window for candidate genes.

Potentially pleiotropic effects of QTLs were observed in a number of regions of different turkey chromosomes. A QTL for PBM was found on chromosome 22 near the QTL for BW and the QTL for the growth curve traits which could probably be explained by a pleiotropic effect of this QTL. In our study, PBM was recorded as a single trait, combining *P*. major and *P*. minor instead of measuring *P*. major and *P*. minor as two separate traits as suggested by Ankra-Badu et al. [36] on chicken who suggested that *P*. major and *P*. minor should be treated separately because these traits were found to be influenced by different QTL [36].

QTL for the breast yield traits, BrL and BrW, were found co-located on chromosome 28 which also harbored QTL for growth traits BW120 and As_wt_, all within a range of 16 cM. These results fit expectations that were based on the high genetic correlation among BrL and BrW with BW traits and As_wt _[5].

No significant QTL were detected for pHu and a*. Some regions on chromosomes 1, 4, 5, 16 (pHu) and 2, 3, 6 (a*) did show an effects on these two traits (pHu and a*) but the observed F-value for these region did not surpass the threshold (Additional files 1 &2). Given the high genetic correlation between PDL and pHu [5], QTL for pHu may have been expected on at least a part of the same chromosomes where QTL for PDL were detected. This lack of concordance may indicate that partially different sets of genes are involved in the control of these traits and/or that there were differences in power to detect QTL for these traits.

A QTL for L* was found in the same region as QTL for BW traits on chromosome 8. Similar to the breast yield traits, L* also had high genetic correlation with BW traits [5] which can be interpreted as an indication towards a pleiotropic nature of this QTL on chromosome 8.

Quality of meat is of interest to breeders and the identification of QTLs, markers and genes associated with meat characteristics would be of great value to improve the meat quality traits which are shown to have reasonable heritabilities (0.09-0.30) in turkeys [5]. In the present study, significant QTL for meat color trait (b*) were detected on three different chromosomes (3, 12, and 26) and suggestive QTL on two additional chromosomes (4 and 21). QTL for PDL were also found on two of these chromosomes (3 and 12). The QTL for PDL on chromosome 3 is, however, located at a distance from the QTL for b* while on chromosome 12, the QTL for PDL was observed in the same region as the QTL for b*. These results are also in agreement with the high genetic correlation between b* and PDL [5].

## Conclusion

A large number of QTL were detected across the turkey genome, which affected growth, breast yield and meat quality traits. Pleiotropic effects or close linkages between QTL were suggested for several of the chromosomal regions. The comparative analysis regarding the location of QTL on different turkey and the syntenic chicken chromosomes, in combination with their association with phenotype revealed signs of functional conservation between these species.

## Authors' contributions

MLA analyzed the data. MLA and JWMB wrote the paper and all other authors gave suggestions and comments for the improvement of paper. All authors read and approved the final manuscript.

## Supplementary Material

Additional file 1 **QTL (data) regions affecting growth, breast yield and meat quality traits mapped on different turkey chromosomes**. Details of QTL regions from all turkey chromosomes with F-statistics using chromosome wide F-statistics threshold. Click here for file

Additional file 2 **QTL (figures) regions affecting growth, breast yield and meat quality traits mapped on different turkey chromosomes**. Peaks showing QTL on different turkey chromosomes with an effect on growth curve, breast yield, body weight and meat quality traits. Click here for file

Additional file 3 **Comparative QTL mapping between turkey and chicken and predicted underlying genes**. Data file with comparative data for QTL positions on different turkey chromosomes with the projection of these positions on different chicken chromosomes. Click here for file

Additional file 4 **List of genes found within the selected window across the significant QTL positions**. This file contains the names of underlying genes within the range of ± 500 Kb (Ten largest chromosomes) and ± 100 Kb (remaining chromosomes) from the projected QTL positions at the different turkey and chicken chromosomes. Click here for file
