# Supplementary material for: Whole genome QTL mapping for growth, meat quality and breast meat yield traits in turkey
Source: BMC Genet. 2011 Jul 11;12:61. doi: 10.1186/1471-2156-12-61 (PMC3142527; doi:10.1186/1471-2156-12-61)
Supplement: Additional file 1 — QTL (data) regions affecting growth, breast yield and meat quality traits mapped on different turkey chromosomes. Details of QTL regions from all turkey chromosomes with F-statistics using chromosome wide F-statistics threshold. [file 1471-2156-12-61-S1.DOC]

**Mapping of QTL at the different turkey chromosomes**

**Growth Curve Traits:** Asymptotic weight (As_wt_), Inflection time (t_mid_) and Scaling Constant (Scale**)**


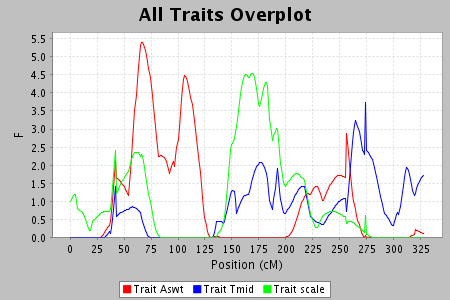

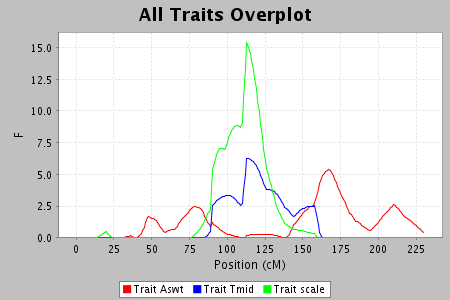

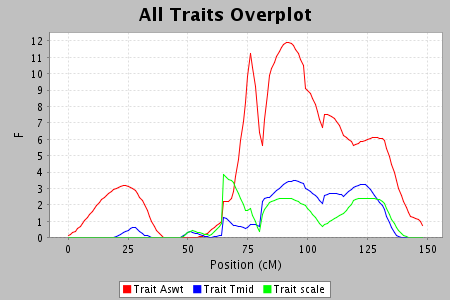

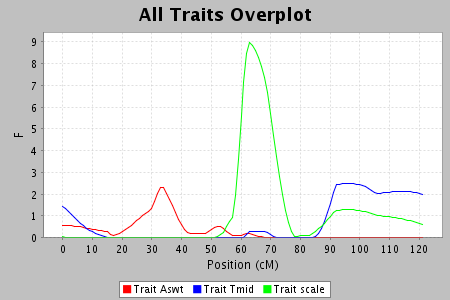

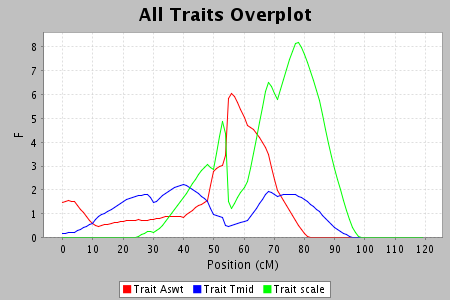

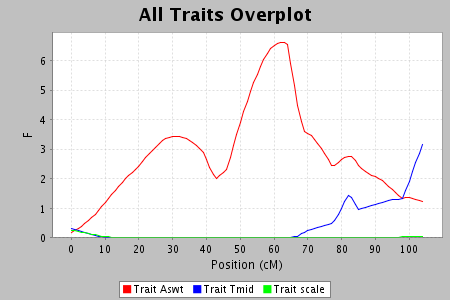

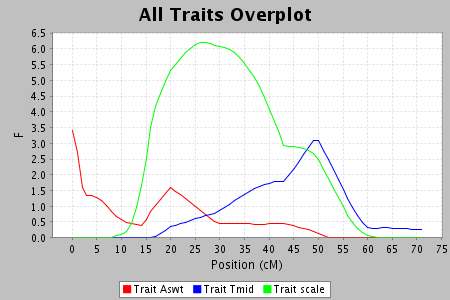

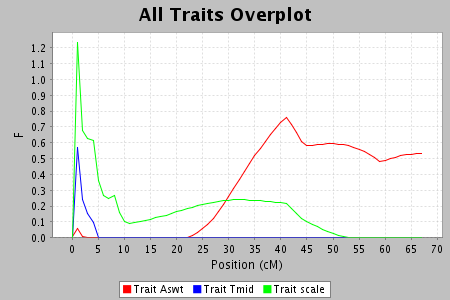


**MGA1 MGA2 MGA3 MGA4**

**MGA5 MGA6 MGA7 MGA8**


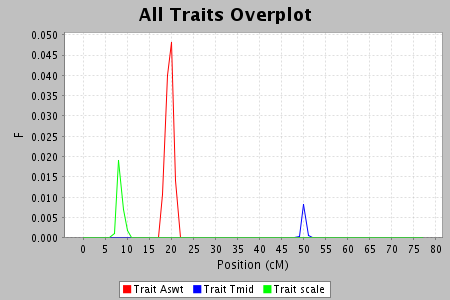

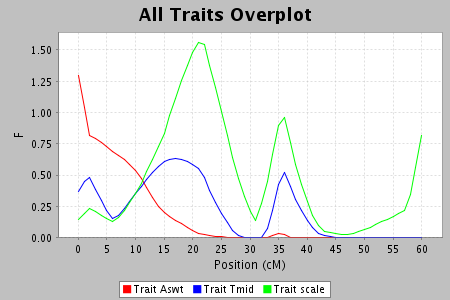

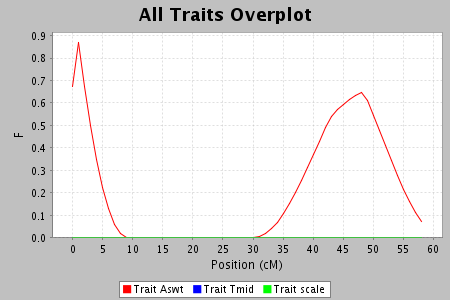


**MGA9 MGA10 MGA11 MGA12**


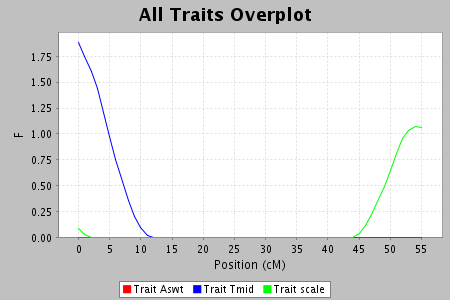


***PIT1***

***AFABP***

***PRKAG3***

***GDF8***

***IGF2***


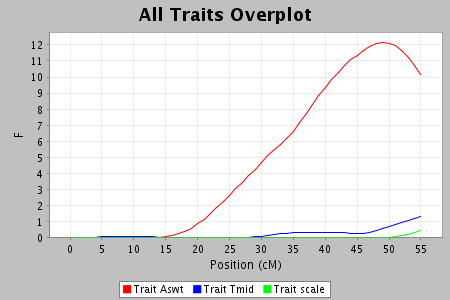

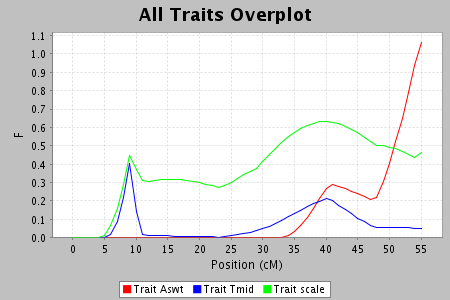

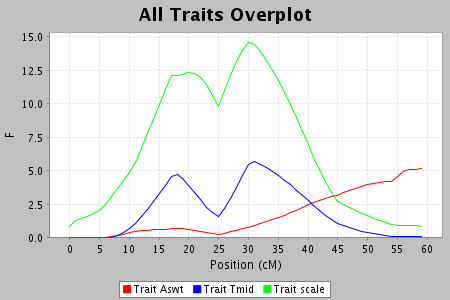

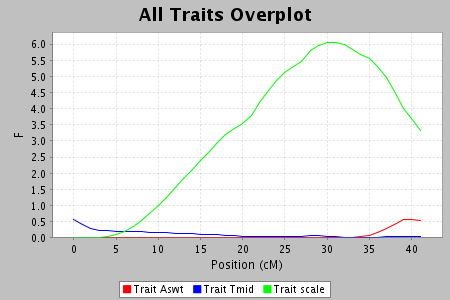

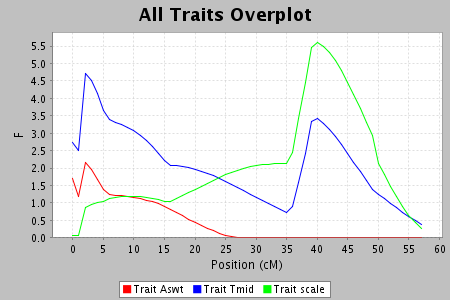

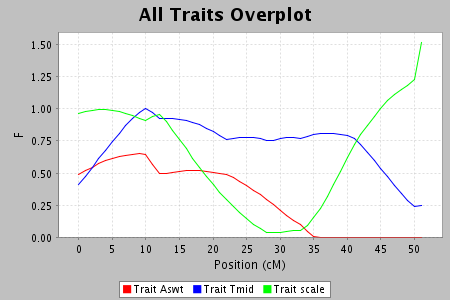

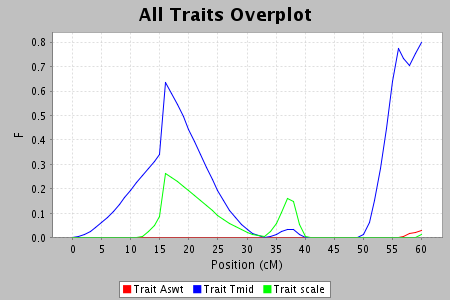

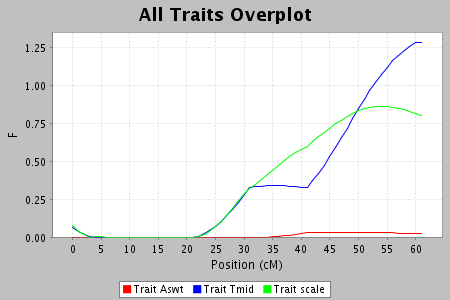

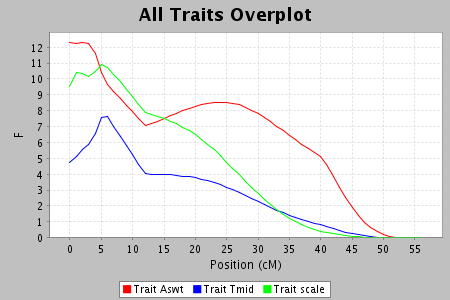

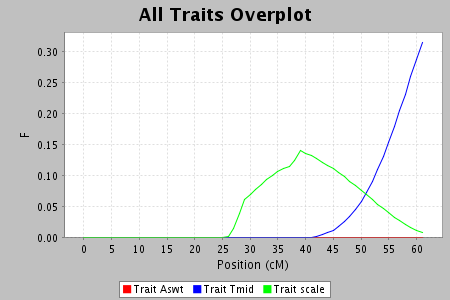

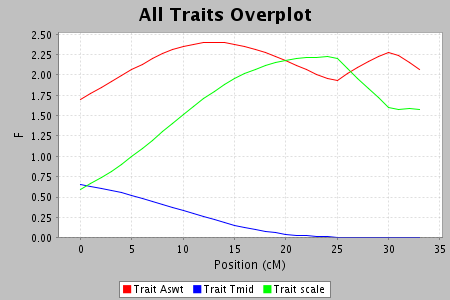

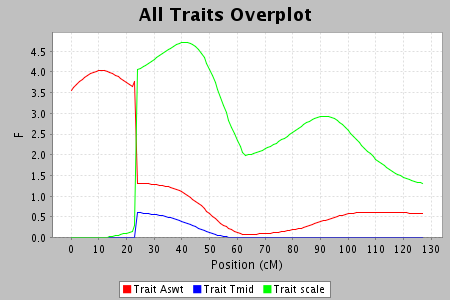


**MGA13 MGA14 MGA15 MGA16**

**MGA17 MGA19 MGA20 MGA21**

**MGA22 MGA23 MGA24 MGA25**


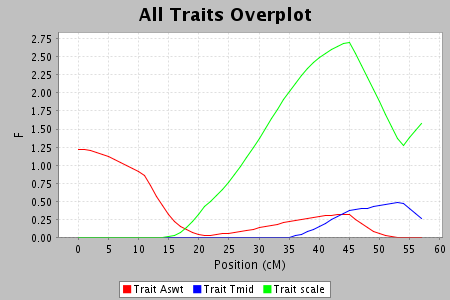

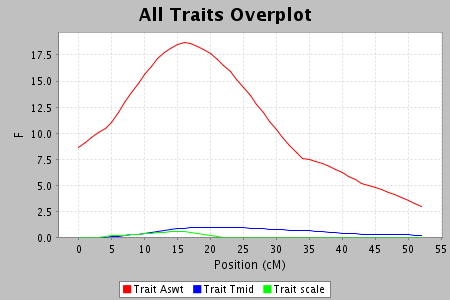

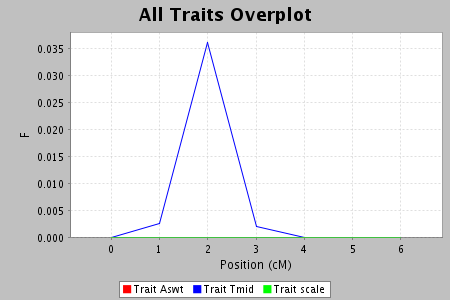


**MGA26 MGA28 MGA30**

**Breast Yield Traits:** Breast Length (BrL), Breast Width (BrW) and Percent Breast Meat (PBM)


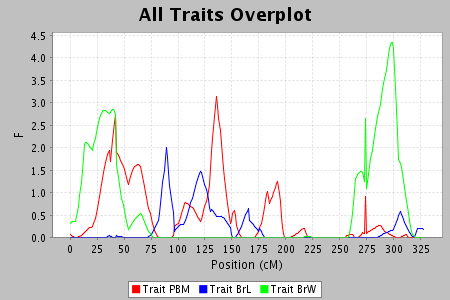

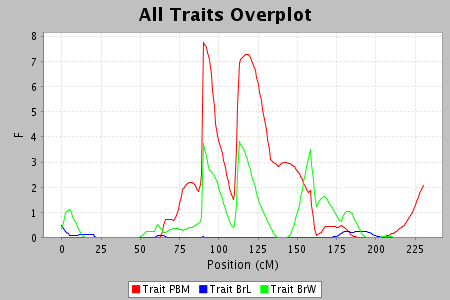

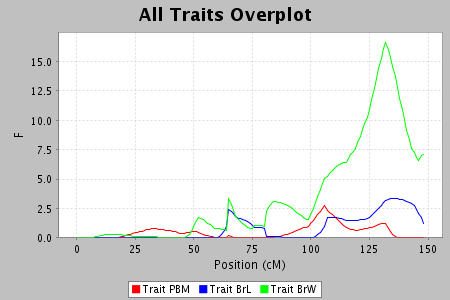

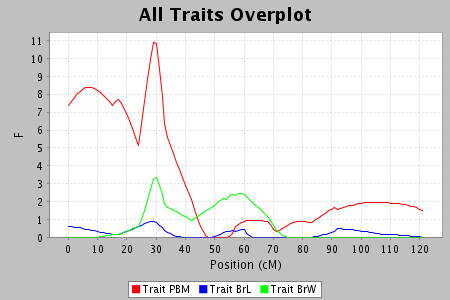

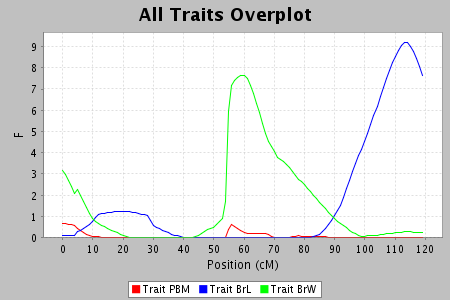

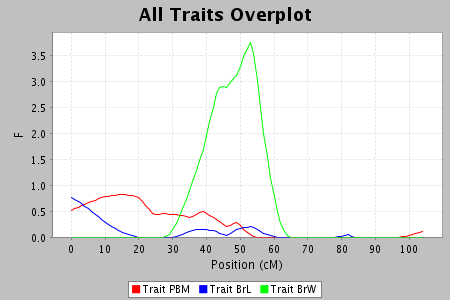

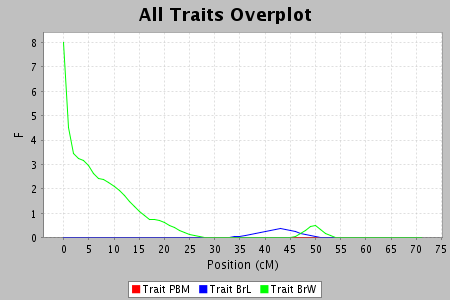

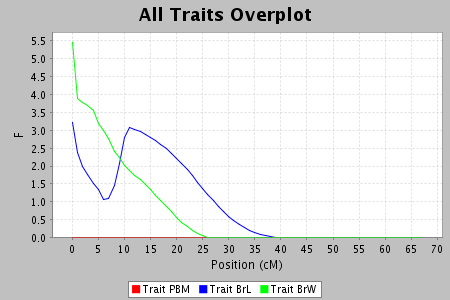


**MGA1 MGA2 MGA3 MGA4**

**MGA5 MGA6 MGA7 MGA8**

***AFABP***

***PIT1***

***GDF8***

***PRKAG3***

***IGF2***


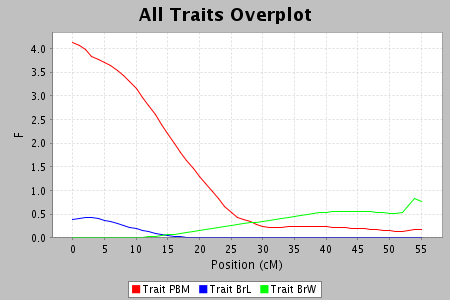

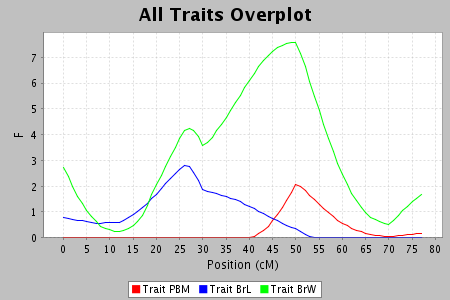

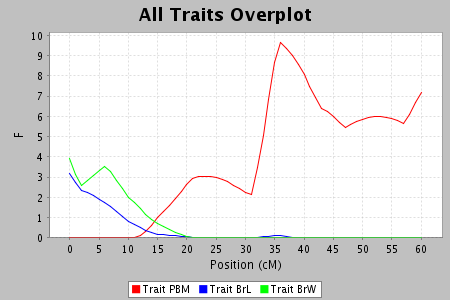

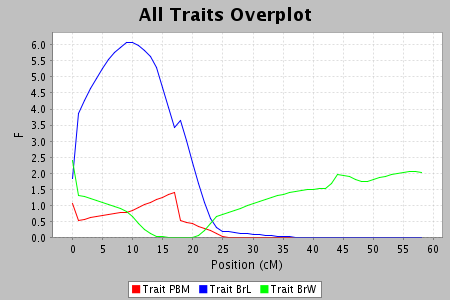

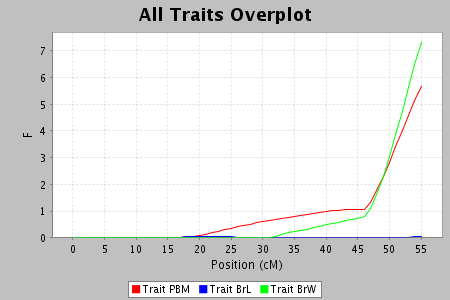

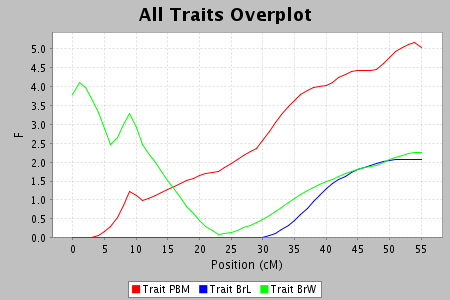

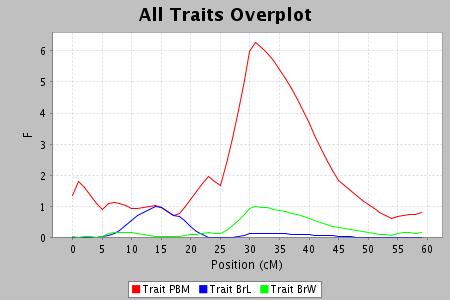

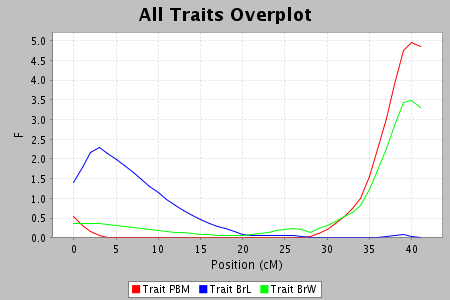

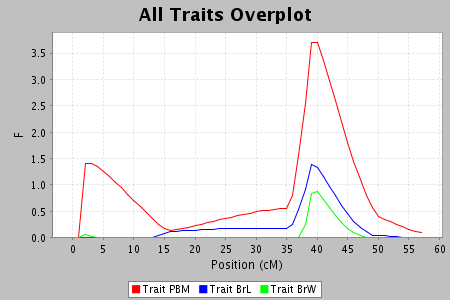

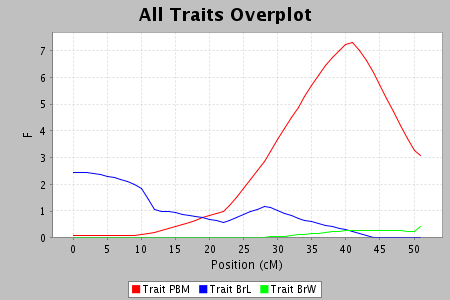

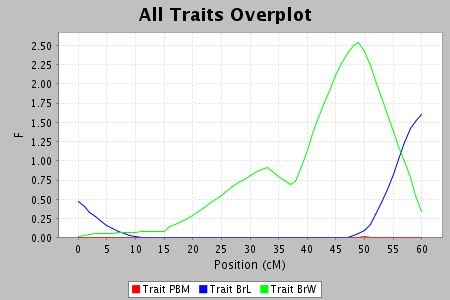

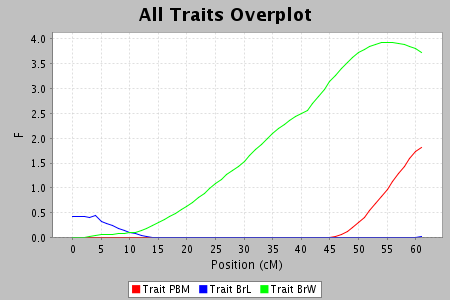


**MGA9 MGA10 MGA11 MGA12**

**MGA13 MGA14 MGA15 MGA16**

**MGA17 MGA19 MGA20 MGA21**


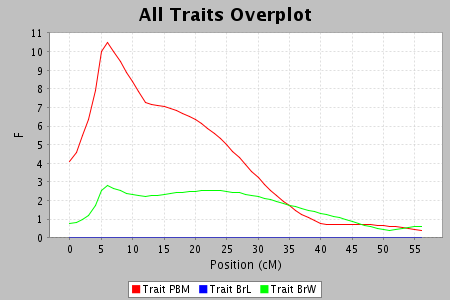

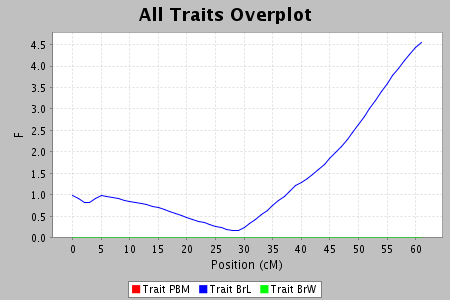

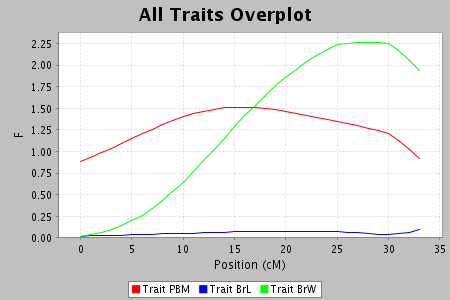

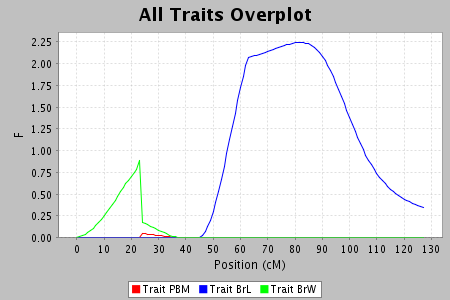


**MGA22 MGA23 MGA24 MGA25**


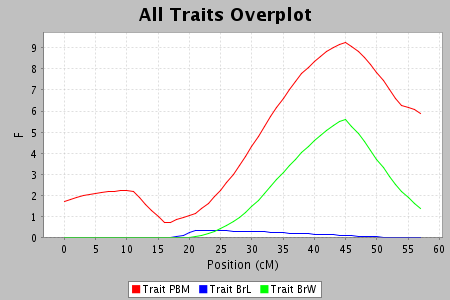

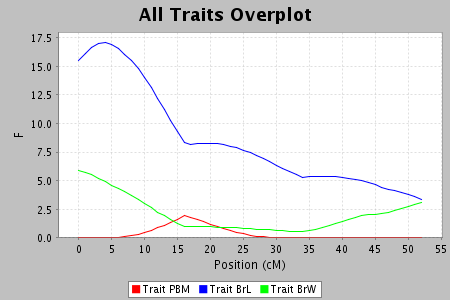

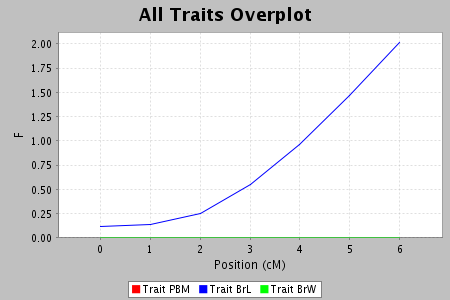


**MGA26 MGA28 MGA30**

**Body Weight (BW) Traits:** Day old BW(BW01), Body Weight at day 17 (BW17), Body Weight at day 40 (BW40), Body Weight at day 60 (BW60), Body Weight at day 80 (BW80), and Body Weight at day 120 (BW120).


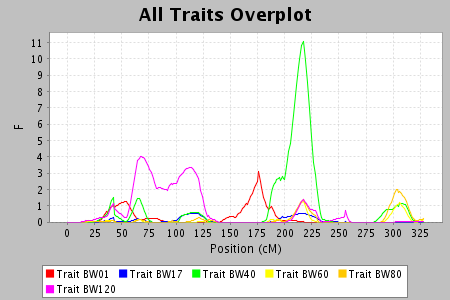

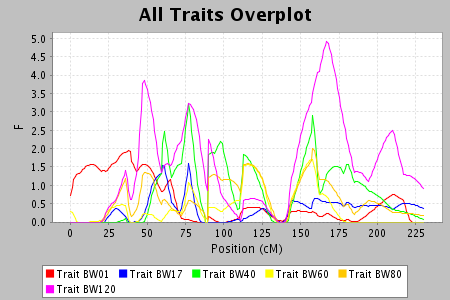

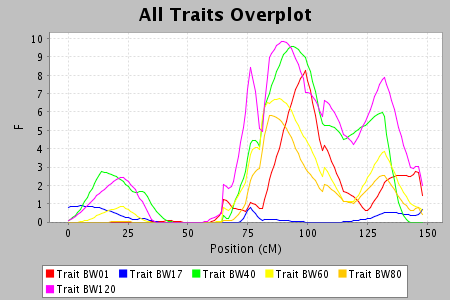

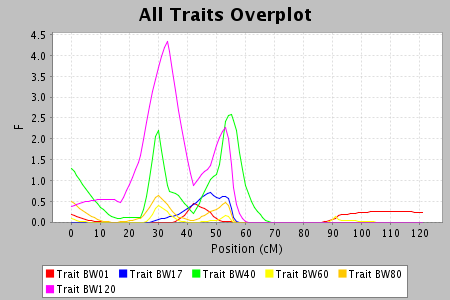

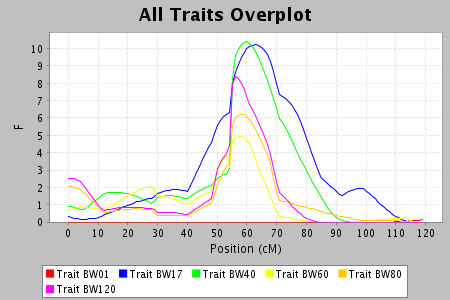

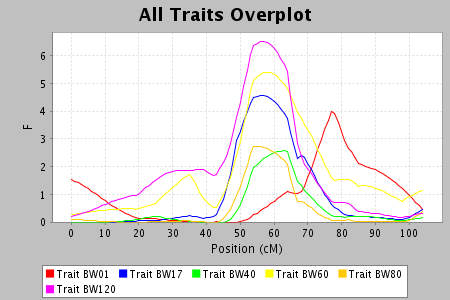

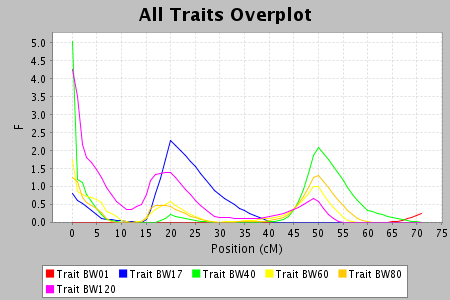

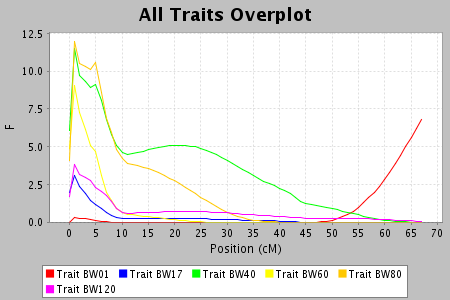

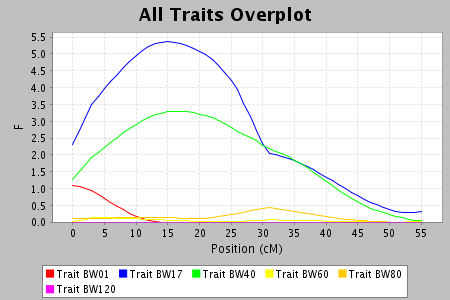

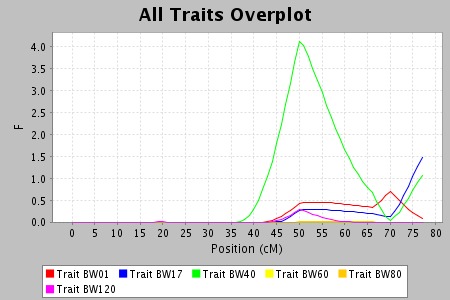

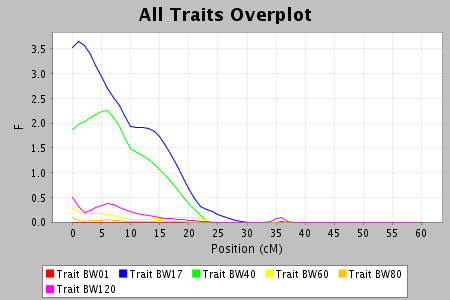

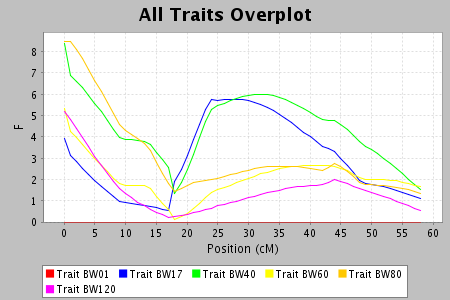


**MGA1 MGA2MGA3 MGA4**

**MGA5 MGA6MGA7 MGA8**

**MGA9 MGA10 MGA11 MGA12**

***PIT1***

***AFABP***

***IGF2***

***PRKAG3***

***GDF8***


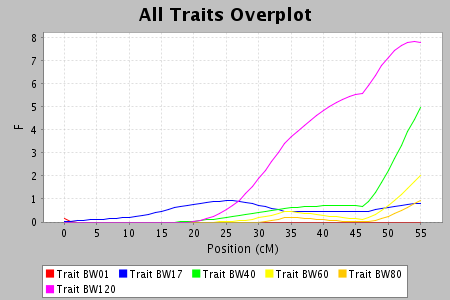

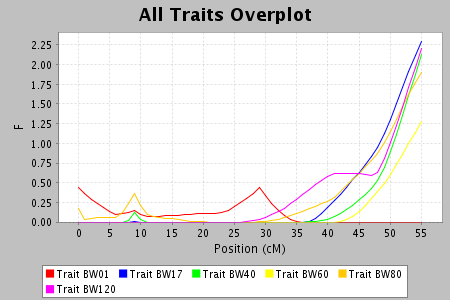

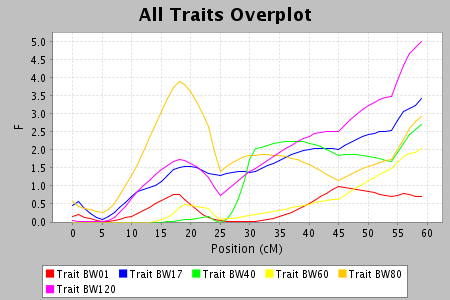

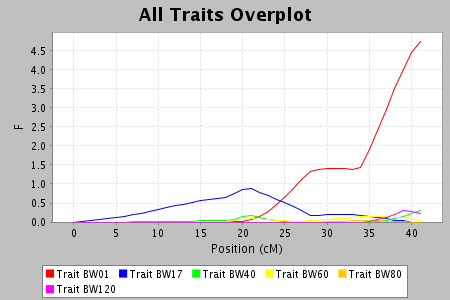

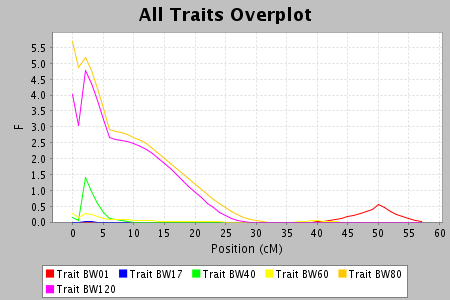

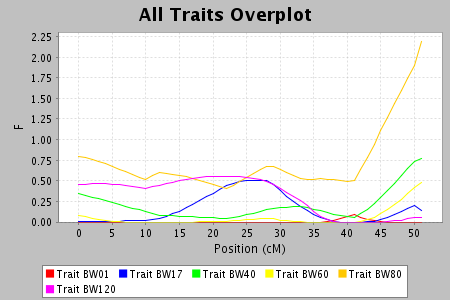

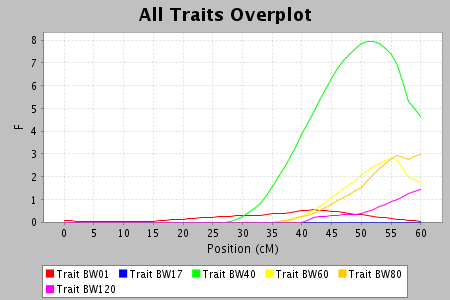

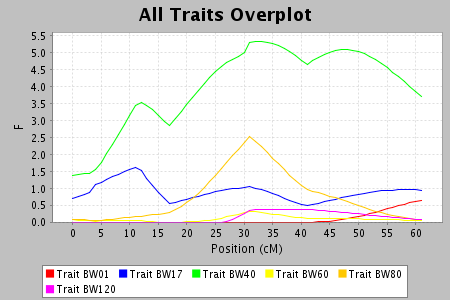

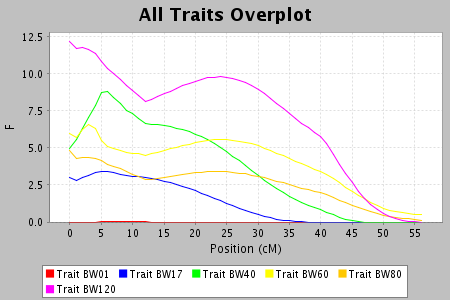

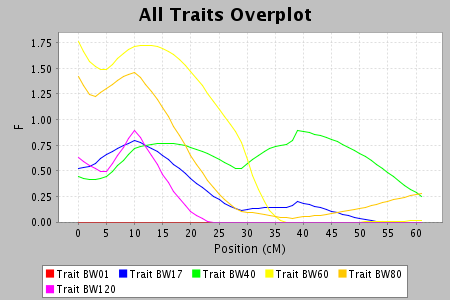

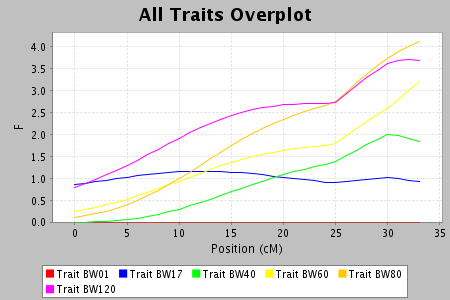

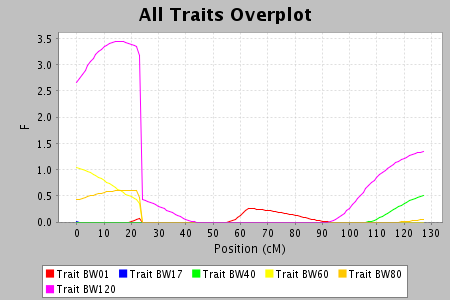


**MGA13 MGA14 MGA15 MGA16**

**MGA17 MGA19 MGA20 MGA21**

**MGA22 MGA23 MGA24 MGA25**


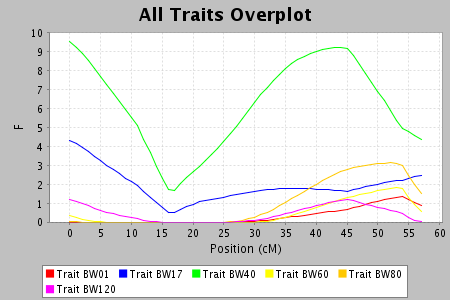

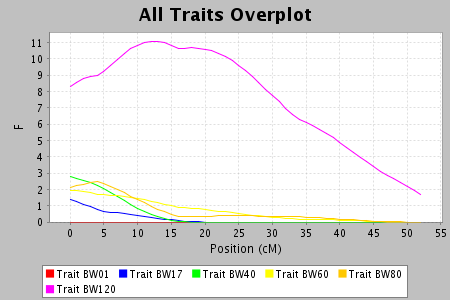

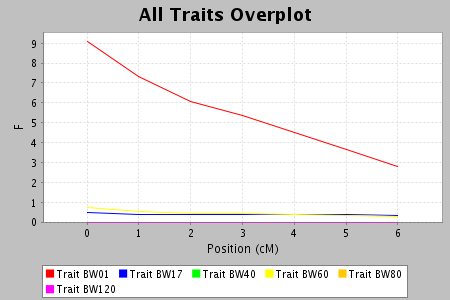


**MGA26 MGA28 MGA30**

**Meat Quality Traits:** Percent Drip Loss(PDL), Ultimate pH (pHu), Lightness (L*), Redness (a*) and Yellowness(b*).


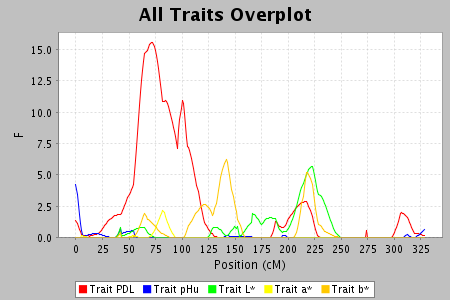

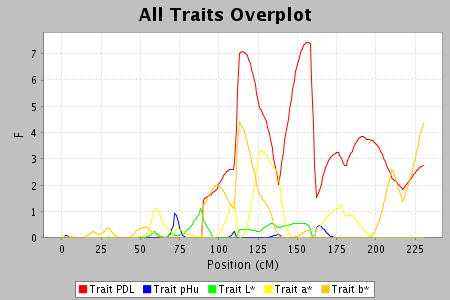

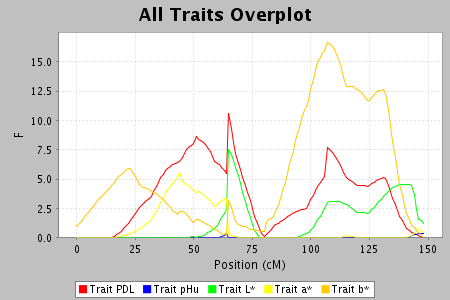

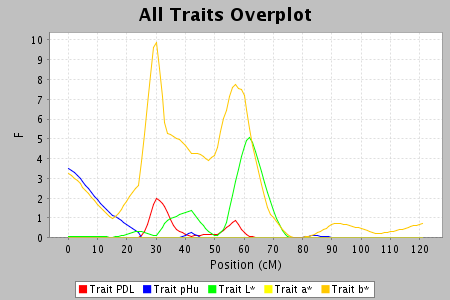

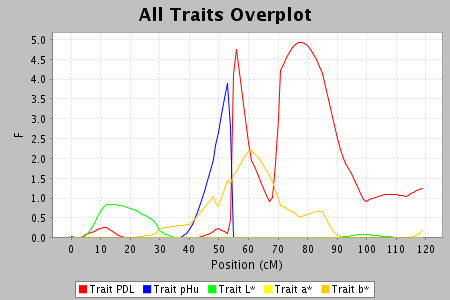

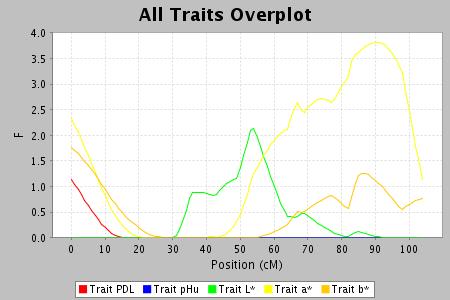

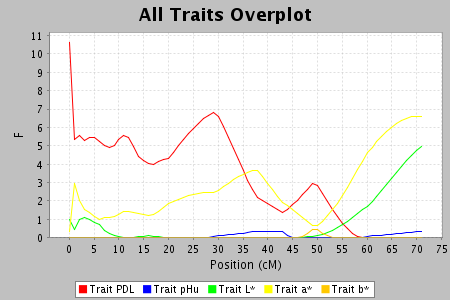

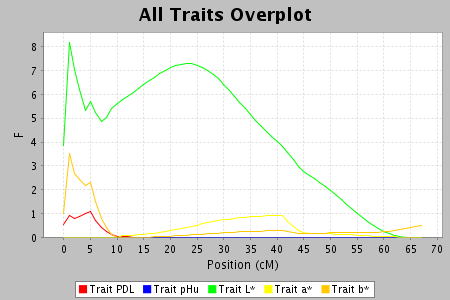


**MGA1 MGA2 MGA3 MGA4**

**MGA5 MGA6 MGA7 MGA8**

***PIT1***

***AFABP***

***IGF2***

***PRKAG3***

***GDF8***


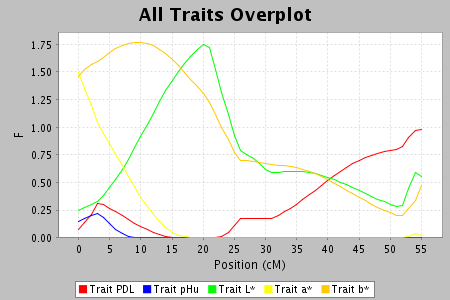

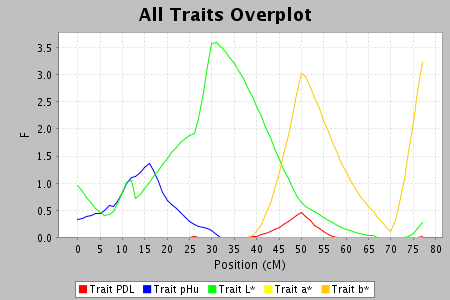

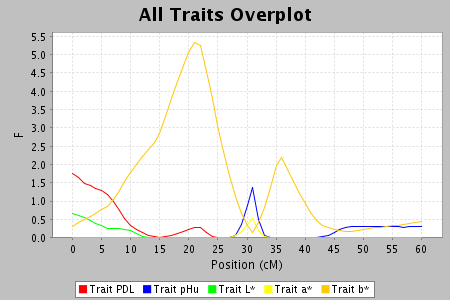

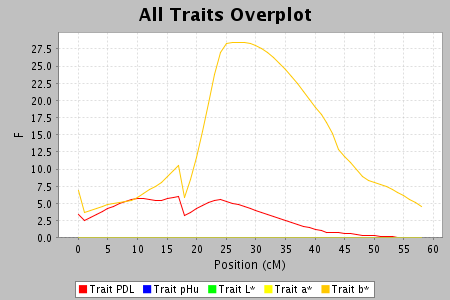

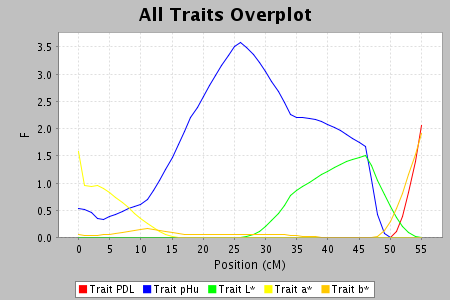

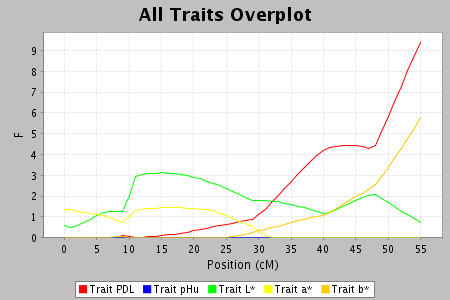

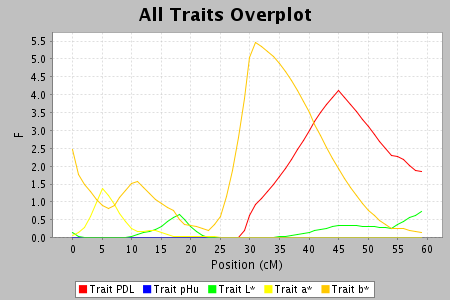

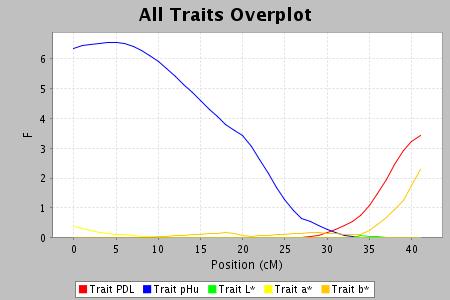

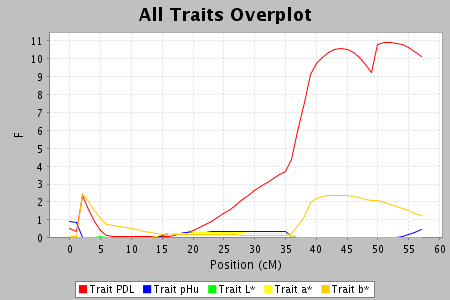

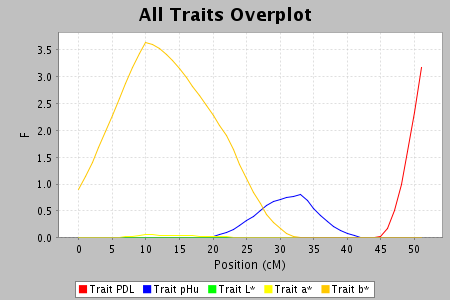

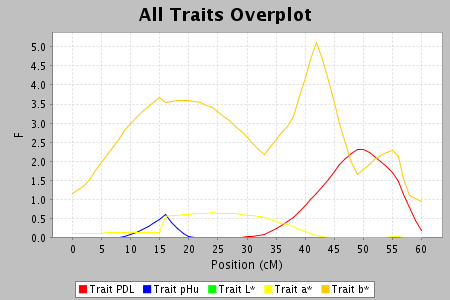


**MGA9 MGA10 MGA11 MGA12**

**MGA13 MGA14 MGA15 MGA16**

**MGA17 MGA19 MGA20 MGA21**

**MGA22 MGA23 MGA24 MGA25**

**MGA26 MGA28 MGA30**

**MGA28**

**MGA8**

**MGA5**

**MGA22**

**MGA12**

**MGA3**

**MGA7**

**MGA7**

***GDF8***

***GDF8***

Effect of SNPs selected from growth gene and F-value reduces to half without the effect of SNPs.
